# Foreign Correspondence

**Published:** 1890-05

**Authors:** 


### Foreign Correspondence.

To the Editor:

Having just returned from a trip through New Zealand, I enclose a report of the first annual meeting of the New Zealand Dental Association, which was formed at a conference of delegates held in Wellington last July.

"The sittings were held at the Criterion, and occupied two days. The following gentlemen were present: Messrs. Boot, Hunter, Throp, J. P. Armstrong, F. Armstrong, Robinson, and Hewitt, of Dunedin; Messrs. Thomas and Merewether, Christchurch; E. Cox, Auckland; J. Goodwin Cox, Timaru; and Thompson, Invercargill. Apologies were read from Messrs. Rawson, Hoby, and others unable to attend.

"Mr. Boot (president of the Association) occupied the chair, and welcomed the members to Dunedin.

"At the sittings of Thursday, after receiving reports from branch representatives, the by-laws of the Association were reconsidered and confirmed, the first only of these evoking prolonged discussion. The question as to whether membership should be thrown open to all registered dentists, without any condition to the mode of conducting practice, was fully discussed.

"The sitting of Friday morning was devoted to the consideration of the 'code of dental ethics.' In compliance with the request of the Wellington Conference, Mr. E. Cox submitted to the meeting a paper upon this subject, dealing with the obligations of the dental practitioner in relation to his profession, to his patients, to his *confrères*, to the medical profession, and to the general public. With the vote of thanks Mr. Cox was desired to revise his paper for publication with the report of the proceedings of the meeting. The afternoon sittings considered the legal opinions received from Sir Robert Stout, and the cases of alleged violation of provisions of the Dentist's Act already open to litigation.

"A vote of thanks to the president and secretary, and the decision to hold the next annual meeting at Christchurch, closed a very cordial and successful conference.

"Alfred Burne.

"Sydney, Australia."

